# In-gel digestion coupled with mass spectrometry (GeLC-MS/MS)-based salivary proteomic profiling of canine oral tumors

**DOI:** 10.1186/s12917-020-02550-w

**Published:** 2020-09-14

**Authors:** Sekkarin Ploypetch, Sittiruk Roytrakul, Narumon Phaonakrop, Suthathip Kittisenachai, Kantinan Leetanasaksakul, Sirinun Pisamai, Chanin Kalpravidh, Anudep Rungsipipat, Gunnaporn Suriyaphol

**Affiliations:** 1 Biochemistry Unit, Department of Physiology, Faculty of Veterinary Science, Chulalongkorn University, 39 Henri-Dunant Road, Wangmai, Pathumwan, Bangkok, 10330 Thailand; 2 Companion Animal Cancer Research Unit, Faculty of Veterinary Science, Chulalongkorn University, 39 Henri-Dunant Road, Wangmai, Pathumwan, Bangkok, 10330 Thailand; 3 Proteomics Research Laboratory, National Center for Genetic Engineering and Biotechnology, National Science and Technology Development Agency, 113 Thailand Science Park, Phahonyothin Road, Khlong Nueng, Khlong Luang, Pathum Thani, 12120 Thailand; 4 Department of Surgery, Faculty of Veterinary Science, Chulalongkorn University, 39 Henri-Dunant Road, Wangmai, Pathumwan, Bangkok, 10330 Thailand; 5 Department of Pathology, Faculty of Veterinary Science, Chulalongkorn University, 39 Henri-Dunant Road, Wangmai, Pathumwan, Bangkok, 10330 Thailand

**Keywords:** Dog, In-gel digestion coupled with mass spectrometry (GeLC-MS/MS), Oral tumors, Tumor protein p53 (p53), Protein tyrosine phosphatase non-receptor type 5 (PTPN5)

## Abstract

**Background:**

Various types of oral tumors, either benign or malignant, are commonly found in dogs. Since saliva directly contacts the tumors and saliva collection is non-invasive, easily accessible and cost effective, salivary biomarkers are practical to be used for the diagnosis and/or prognosis of these diseases. However, there is limited knowledge of protein expression in saliva for canine oral tumors. The present study aimed to investigate novel biomarkers from the salivary proteome of dogs with early- and late-stage oral melanoma (EOM and LOM, respectively), oral squamous cell carcinoma (OSCC), benign oral tumors (BN), and periodontitis and healthy controls (CP), using an in-gel digestion coupled with mass spectrometry (GeLC-MS/MS). The relationships between protein candidates and chemotherapy drugs were explored and the expression of potential biomarkers in saliva and tissues was verified by western blot analysis.

**Results:**

For saliva samples, increased expression of protein tyrosine phosphatase non-receptor type 5 (PTPN5) was shown in all tumor groups compared with the CP group. Marked expression of PTPN5 was also observed in LOM and OSCC compared with that in BN and EOM. In addition, tumor protein p53 (p53), which appeared in the PTPN5–drug interactions, was exhibited to be expressed in all tumor groups compared with that in the CP group. For tissue samples, increased expression of p53 was shown in LOM compared with the control group.

**Conclusion:**

PTPN5 and p53 were proposed to be potential salivary biomarkers of canine oral tumors.

## Background

Head and neck tumors comprise approximately 7% of all tumors in dogs. Among these, oral melanoma (OM) and oral squamous cell carcinoma (OSCC) are most commonly found [1]. The tumor, node and metastasis (TNM) classification of tumors in the oral cavity are described. Stages I and II refer to tumors with ≤2 cm and 2 to < 4 cm, respectively, defined as early clinical stages with no metastasis, whereas stage III refers to a tumor with ≥4 cm and/or lymph node metastasis and stage IV refers to a tumor with distant metastasis. The latter two are defined as late clinical stages and are most frequently observed in the animal hospital owing to the difficulty in routinely examining tumors in dogs’ mouths [2–4]. After surgical resection, patients with late clinical stage are normally treated with chemotherapy drugs such as carboplatin, a derivative of the anticancer drug cisplatin, doxorubicin (or Adriamycin**®**), cyclophosphamide and piroxicam. With a high rate of metastasis and recurrence of oral cancer, novel biomarkers are important for early clinical diagnosis, screening and prognosis of the diseases [5]. Saliva proteins have high potential to be appropriate biomarkers because saliva makes direct contact with an oral mass, and saliva collection is non-invasive and not difficult to manipulate [6]. Novel salivary proteome biomarkers have been discovered in human oral tumors [7–10]. However, in dogs with oral diseases, the evidence of proteomics in saliva is still limited [6]. The present study aimed to search for novel suitable biomarkers in saliva of dogs with early- and late-stage oral melanoma (EOM and LOM, respectively), oral squamous cell carcinoma (OSCC), benign oral tumors (BN), periodontitis (P) and healthy controls (C) (CP group), using in-gel digestion coupled with mass spectrometry (GeLC-MS/MS). Associations of disease-related proteins with the chemotherapy drugs cisplatin, cyclophosphamide, piroxicam and doxorubicin were exhibited. The candidate protein expressions in saliva and tissues were affirmed by western blot analysis.

## Results

### GeLC-MS/MS results

**Fig. 1:**
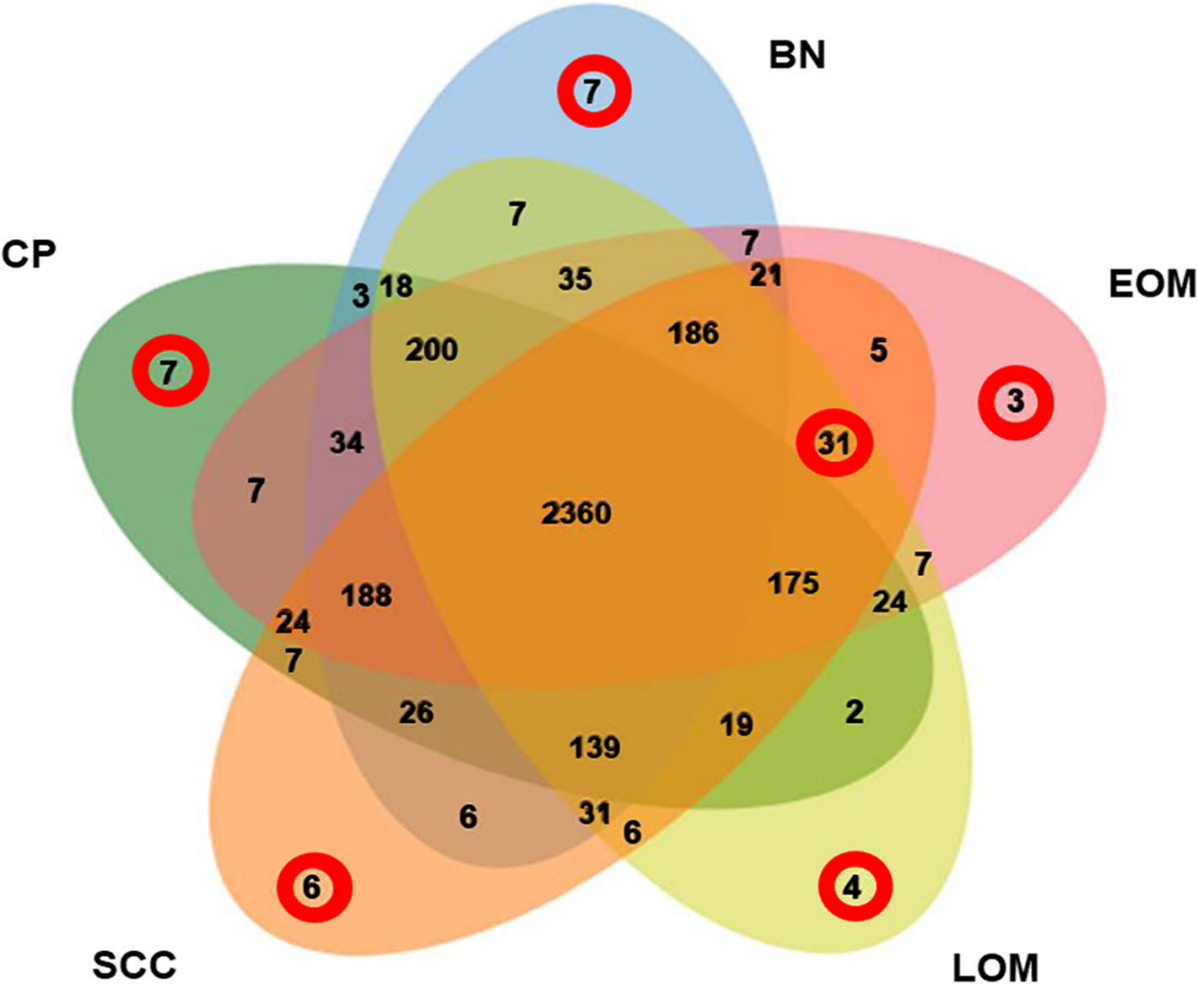
Venn diagram of proteins differentially expressed in early-stage OM (EOM), late-stage OM (LOM), oral squamous cell carcinoma (OSCC), benign oral tumors (BN) and normal and periodontitis (CP). Circles indicate overexpressed proteins uniquely found in each group and commonly found in all cancerous groups

**Fig. 2:**
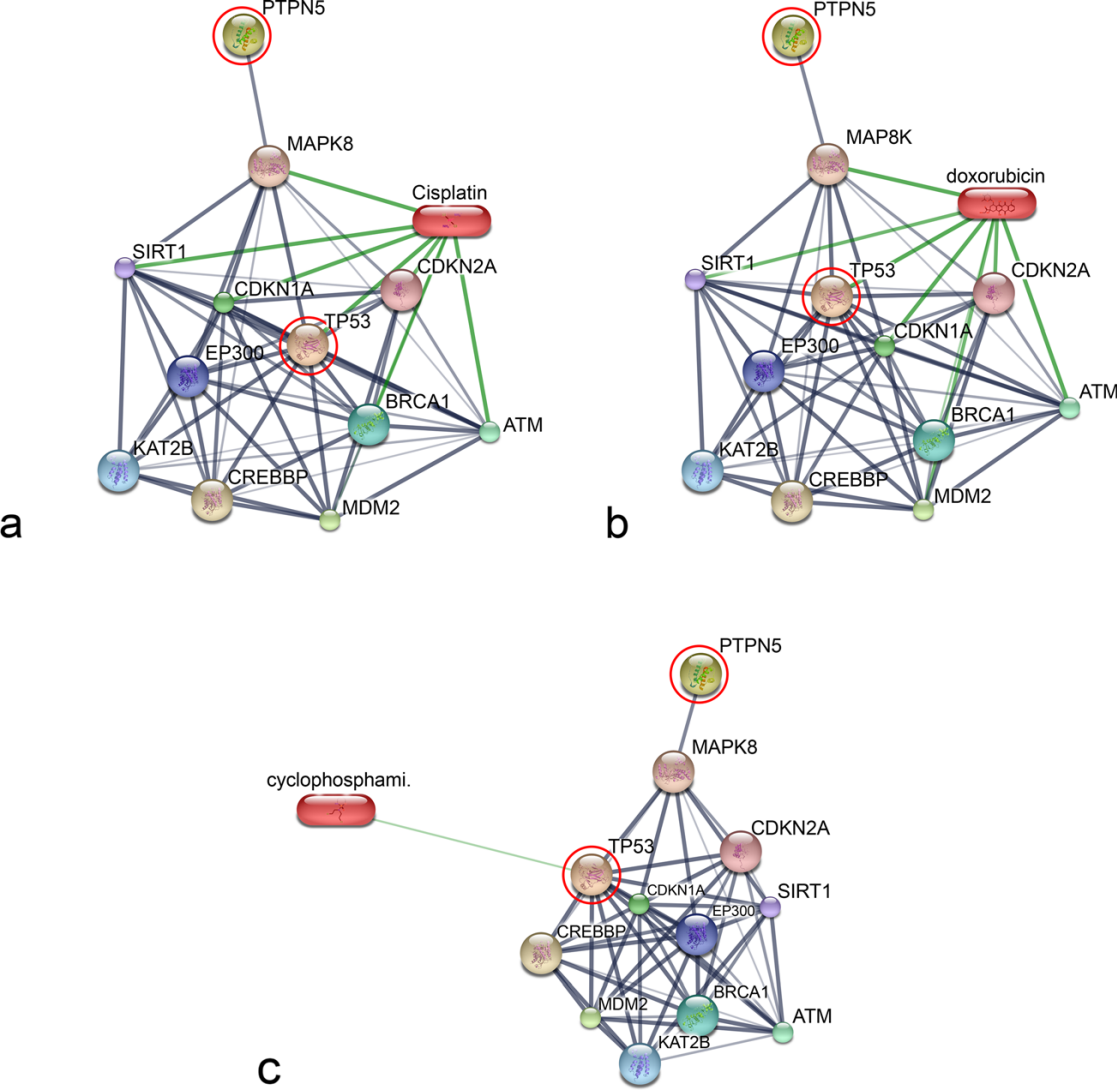
Involvement of tyrosine-protein phosphatase non-receptor type 5 (PTPN5) and tumor protein p53 (TP53) in networks of protein chemotherapy drug interactions, cisplatin and doxorubicin, analysed by Stitch, version 5.0; **a** Interactions of PTPN5 and TP53 with cisplatin; **b** Interactions of PTPN5 and TP53 with doxorubicin; **c** Interactions of PTPN5 and TP53 with cyclophosphamide. Red circles: PTPN5 and TP53. Abbreviations: ataxia telangiectasia mutated (ATM), breast cancer 4721, early onset (BRCA1), cyclin-dependent kinase inhibitor 1A (p21, Cip1) (CDKN1A), cyclin-. 34,473 dependent kinase inhibitor 2A (CDKN2A), CREB binding protein (CREBBP), E1A binding 474 protein p300 (EP300), K (lysine) acetyltransferase 2B (KAT2B), mitogen-activated protein kinase 4758 (MAPK8), Mdm2 (MDM2) and sirtuin 1 (SIRT1).

A total of 3726 proteins were identified. The distribution of the individual and overlapped proteins in EOM, LOM, OSCC, BN and CP groups was illustrated by a Venn diagram (Fig. 1). In addition, the molecular function, biological process, cellular component and the relative expression levels of the proteins uniquely expressed in each group and commonly expressed in all cancerous groups was analysed using the PANTHER software tools (Tables 1 and 2 and Supplementary Table S1). For the networks of protein–protein and protein–chemotherapy drug interactions, analysed by the Stitch program, version 5.0, edge confidence scores demonstrated the strength of the interactions at the functional level. Pathways with high edge confidence scores (> 0.700) were presented as thick lines. The associations of protein tyrosine phosphatase non-receptor type 5 (PTPN5) and tumor protein p53 (p53) with cisplatin and doxorubicin drugs were shown. Additionally, the correlation of PTPN5 and cyclophosphamide was demonstrated (Fig. 2). In the present study, increased expression of another protein involved in the SUMOylation process, RanBP2, was noted in a cancerous group (Table 2). RanBP2 regulated translocation of p53, a well-known target of SUMOylation, to the cytoplasm, leading to poor prognosis and prostate cancer progression [11].

**Table 1:** Overexpressed proteins uniquely found in normal controls and periodontitis, benign oral tumors, early-stage oral melanoma, late-stage oral melanoma and oral squamous cell carcinoma based on biological process involvement and protein score

Database	Protein name	Protein ID score	Peptides	Biological process	Subcellular distribution
**Normal controls and periodontitis**					
XP_016007048.1	Semaphorin-4B isoform X1	13.9	QLVASYCPK	1. Negative chemotaxis 2. Semaphorin–plexin signalling pathway	1. Extracellular space 2. Integral component of plasma membrane
XP_011988340.1	Visual system homoeobox 1 isoform X2	16.98	FPGRPLPSAARQK	1. Multicellular organism development 2. Regulation of transcription	1. Nucleus 2. Cytoskeleton
XP_013973434.1	CDK5 regulatory subunit-associated protein 2 isoform X1	12.52	FTNQGKR	Microtubule organizing center	
XP_002689199.3	Olfactory receptor 2 M5	26.19	MCWQVAAMSWAGGAR	Olfaction	Plasma membrane
XP_008048855.1	Potassium voltage-gated channel subfamily Q member 1	34.67	LNIEDFR	1. Potassium ion export across plasma membrane 2. Cellular response to cAMP	1. Endoplasmic reticulum 2. Endosome 3. Plasma membrane
XP_007125871.1	GLIPR1-like protein 1	14.03	AHNEAR	Single fertilization	Plasma membrane
EHB15707.1	Transient receptor potential cation channel subfamily M member 5	26.14	TVAPKSLLFR	Ion transmembrane transport	Plasma membrane
**Benign oral tumors**					
KFO21119.1	Germ cell-less protein-like 1	7.86	KAVAAR	Cell differentiation	Nucleus
XP_004629194.1	Poly [ADP-ribose] polymerase 12	21.09	KLGMSSELVHR	Protein auto-ADP-ribosylation	Nucleus
XP_015289690.1	Lamin tail domain-containing protein 2	8.98	GLLPPMSSGK	Cell population proliferation	1. Cytoskeleton 2. Nucleus
XP_012868232.1	Telomeric repeat-binding factor 2-interacting protein 1	16.48	AEPDPEAAESVEPQTK	1. Negative regulation of DNA recombination at telomere 2. Positive regulation of NF-κB transcription factor activity	Nucleus
XP_012373519.1	Myb-related protein B	16.69	MLPGRYVPGGGVGAR	1. Mitotic cell cycle 2. Regulation of cell cycle	Nucleus
XP_012865682.1	Erythrocyte membrane protein band 4.2	12.59	QWSAVVEDR	1. Cell morphogenesis 2. Hemoglobin metabolic process	Cytoskeleton 1. Cytoplasm 2. Membrane
XP_005371197.1	Long-chain-fatty-acid–CoA ligase ACSBG2	5.91	APGTGFLTEMLR	cell differentiation	
**Early-stage oral melanoma**					
XP_011760132.1	Putative protein SSX6	12.53	GGNMPGPTGCVR	Regulation of transcription, DNA-templated	Nucleus
XP_004326275.1	Bromodomain testis-specific protein-like	14.28	DNAKPMNYDEKR	Chromatin remodelling	Nucleus
XP_006868797.1	Zinc finger protein GLI2-like	16.61	GGSLENSSIPDLSR	Nucleic acid binding	Nucleus
**Late-stage oral melanoma**					
EPQ15807.1	Transformation/transcription domain-associated protein	9.28	AMAILTPAVPAR	1. DNA repair 2. Histone deubiquitination	1. Golgi apparatus 2. Nucleus
XP_009240233.1	Glutathione S-transferase-like	20.93	ARISHILTINK	Glutathione transferase activity	Cytoplasm
XP_011282224.1	Protein FAM186A	32.14	SVEQSFLELLIEEDR	No data	1. Nucleus 2. Cytoplasm
XP_004412391.1	Deleted in lung and oesophageal cancer protein 1	7.49	AGPPKNK	Negative regulation of cell population proliferation	Cytoplasm
**Oral squamous cell carcinoma**					
XP_007944568.1	Ankyrin repeat domain-containing protein 26-like	6.56	ADIKENMVIDMQANCMILXK	Protein interaction	Cytoplasm
XP_012392091.1	Cytohesin-4 isoform X2	9.84	YPGELSSGEAEELQR	Regulation of ARF protein signal transduction	Nucleus
XP_007532207.2	Probable C-mannosyltransferase DPY19L4	17.69	KPKSSGNK	Protein C-linked glycosylation via 2′-alpha-mannosyl-L-tryptophan	Membrane
EHB17858.1	Dynein heavy chain 11, axonemal	3.80	ATSEMR	Determination of left/right symmetry	Cytoskeleton
XP_004275614.1	Fanconi anaemia-associated protein of 100 kDa	7.99	XGMDDR	Interstrand cross-link repair	Nucleus
OBS77059.1	Protein A6R68_16468	7.01	DQVSDDVSVQSSGPNCQR	Regulation of transcription by RNA polymerase II	Nucleus

**Table 2:** Overexpressed proteins commonly found in early-stage oral melanoma, late-stage oral melanoma and oral squamous cell carcinoma based on biological process involvement and protein score

Database	Protein name	Protein ID score	Peptides	Biological process	Subcellular distribution
XP_005376885.1	ATP synthase subunit s, mitochondrial isoform X1	4.77	HQTMLFGK	ATP biosynthetic process	Mitochondria
XP_004411845.1	Carbonic anhydrase 12 isoform X1	33.40	SLHAAAVLLLLCFK	Carbonate dehydratase activity	Integral component of membrane
XP_015354861.1	Cell division cycle-associated protein 2	17.63	RSFCAPTLSSK	Cell cyclecell division	Nucleus
XP_004625867.1	dihydroorotate dehydrogenase (quinone), mitochondrial	17.17	IPIIGVGGVSSGQDAMDK	‘de novo’ UMP biosynthetic process	Mitochondrion inner membrane
XP_014948096.1	Hermansky–Pudlak syndrome 3 protein isoform X1	9.93	ACPPISMDVCALR	Organelle organization,pigmentation	Cytosol
XP_004644982.1	KN motif and ankyrin repeat domain-containing protein 3	14.22	FALNQNLPDLGGSR	Negative regulation of actin filament polymerization	Cytoplasm
XP_008158631.1	Leucocyte immunoglobulin-like receptor subfamily A member 6	3.43	EPAEVEELK	Adaptive immune response	Membrane
XP_003787787.1	Negative elongation factor C/D	7.47	SNFIMMN	Transcription by RNA polymerase II	Nucleus
XP_011285357.1	Neurexin-2-β	13.66	VVVVLGGQGSSG	Neuron cell–cell adhesion signal transduction	Membrane
XP_005629058.1	Origin recognition complex subunit 1 isoform X1	6.66	SRPTPSHPATPRAK	DNA replication,mitotic cell cycle	Nucleus
XP_006896914.1	Phosphoenolpyruvate carboxykinase, cytosolic [GTP] isoform X1	18.32	ARVSQM	Gluconeogenesis	Cytosol
XP_004620060.1	Phospholipase B1, membrane-associated-like	11.55	RMENNSGINFNEDWK	Phospholipase activity	Integral component of membrane
XP_012626009.1	Progesterone receptor isoform X2	17.75	VLLLLNTTR	DNA-binding transcription factor activity	Nucleus
XP_008151988.1	Secernin-2	13.13	QGGITAEAMMDILRDK	Exocytosis	Extracellular exosome
XP_007489730.1	Sodium/iodide cotransporter	6.99	DSKEYPQEVK	Cellular response to cAMP	Membrane
XP_016811442.1	T-box transcription factor TBX18 isoform X2	12.54	MYSGELGPI	DNA-binding transcription factor activity	Nucleus
XP_004045865.1	Uncharacterized protein LOC101132572	12.64	RFTLSLDAPAPTQGVCK	Unknown	Unknown
XP_006190947.1	Zinc finger protein ZIC 3	8.6	THTGKGEGGR	Cell differentiation	Nucleus
XP_011744397.1	28S ribosomal protein S14, mitochondrial	16.97	KNTXLPK	Mitochondrial translational elongation and translation	Mitochondria
XP_007505382.1	3-hydroxyisobutyrate dehydrogenase, mitochondrial isoform X1	8.97	SMASKTPVGFVGLGNMGNPMAK	3-hydroxyisobutyrate dehydrogenase activity	Mitochondria
XP_004448347.1	α-ketoglutarate-dependent dioxygenase alkB homolog 4 isoform X1	7.08	LVSLNLLSSTVLSMSR	Demethylation	Mitochondria
XP_005065718.1	Ankyrin repeat domain-containing protein 34B	20.75	QKALMTTNGPK	Unknown	Nucleus
NP_036833.1	β1 adrenergic receptor	13.02	QGFSSESK	Adenylate cyclase-activating adrenergic receptor signalling pathway	Endosome,plasma membrane
ELK12127.1	Cytochrome b-c1 complex subunit 2, mitochondrial	11.51	DNMAYTGEGLR	Aerobic respiration	Mitochondria
XP_006883886.1	E3 SUMO-protein ligase RanBP2	11.07	LSQSGHMLINLSRGK	centrosome localization	Nucleus
BAD96349.1	Heme oxygenase (decyclizing) 2 variant	11.2	KSSGALEK	Heme oxygenase (decyclizing) 2 variant	Endoplasmic reticulum
OBS70980.1	Pyrroline-5-carboxylate reductase	9.86	LTAFXPAPK	L-proline biosynthetic process	Mitochondria
XP_015976454.1	Laminin subunit α1	15.83	YXNGTWYK	Cell adhesion	Extracellular region or secreted
KFO28259.1	Mitochondrial import receptor subunit TOM20 like protein	10.02	LFSVQMPLAKLPTTGQR	Protein import into mitochondrial matrix	Mitochondria
EAW72809.1	Signal sequence receptor, delta (translocon-associated protein delta), isoform CRA_c	3.09	APTQAPMR	Regulate the retention of ER resident proteins	Endoplasmic reticulum
XP_006865897.1	Tyrosine-protein phosphatase non-receptor type 5	21.9	AEGLRGSHR	Cellular response to cytokine stimulus	Endoplasmic reticulum

### Western blot analysis results

**Fig. 3:**
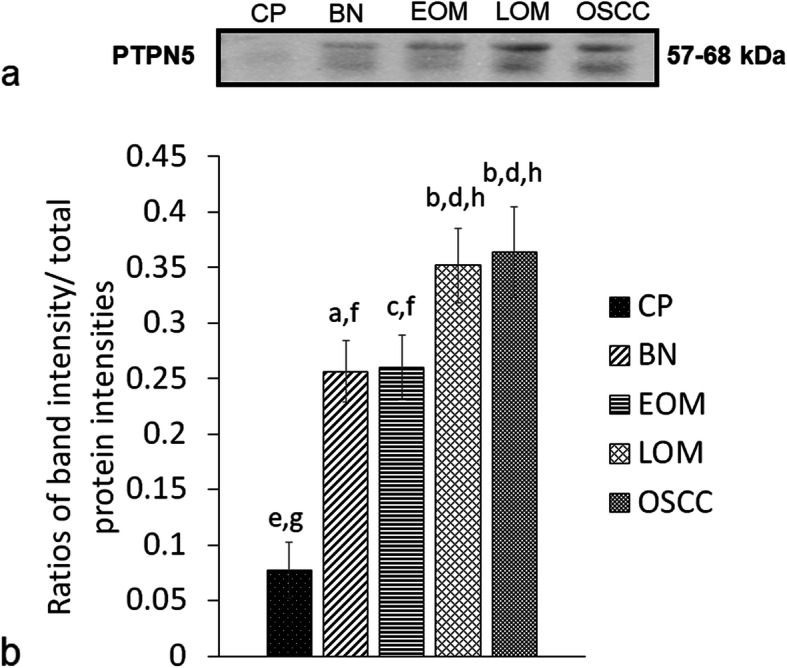
Western blot analysis of salivary tyrosine-protein phosphatase non-receptor type 5 (PTPN5) of dogs with benign oral tumors (BN), early- and late-stage oral melanoma (EOM and LOM, respectively), oral squamous cell carcinoma (OSCC) and periodontitis and normal controls (CP); **a** Representative western blot for PTPN5 at 57–68 kDa; **b** bar graph of ratios of PTPN5 protein intensity to total blotted proteins in each lane in a membrane; a-b and c-d denote a significant difference at *P* < 0.05; e-f denote a significant difference at *P* < 0.001; g-h denote a significant difference at *P* < 0.0001

**Fig. 4:**
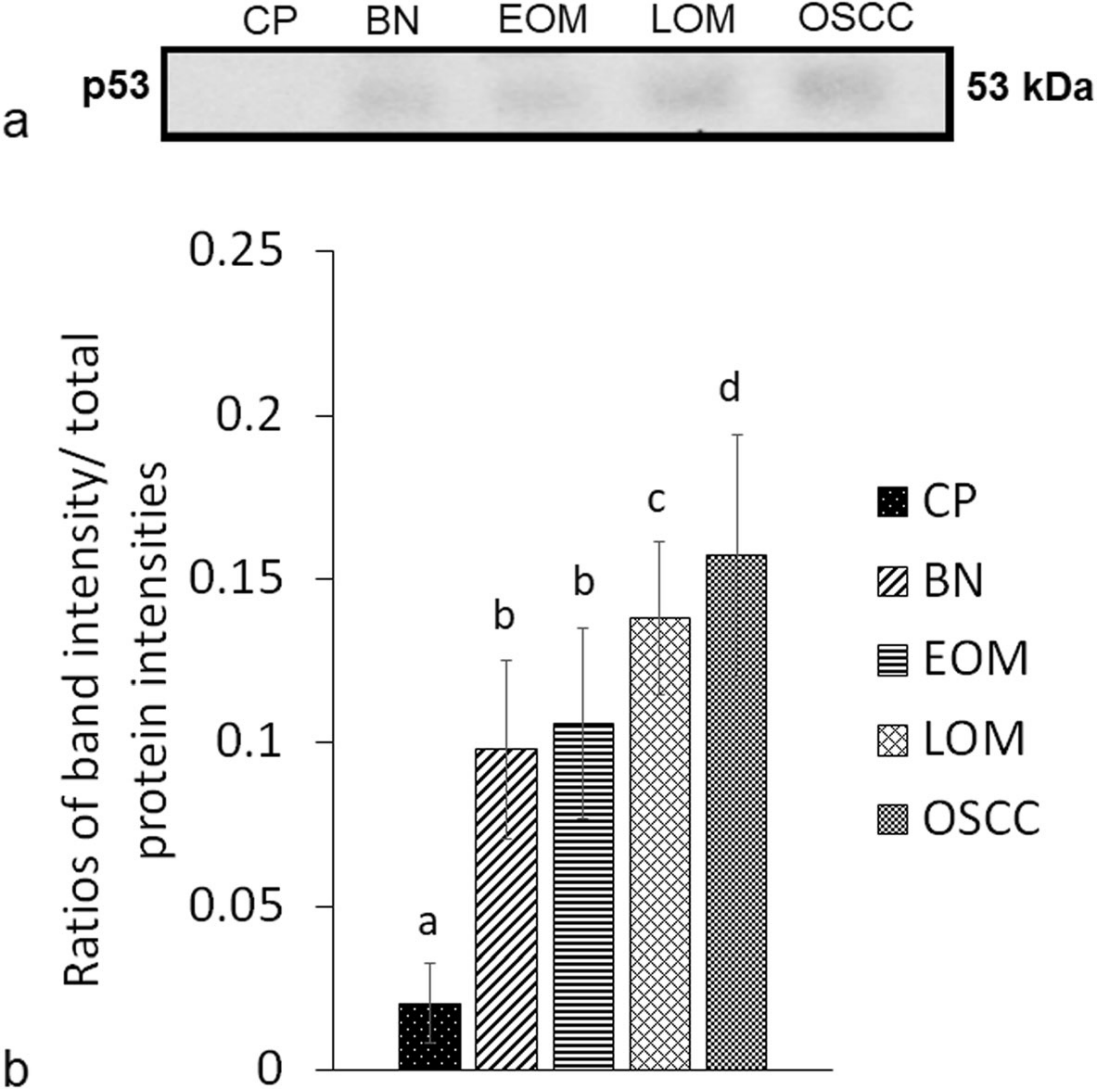
Western blot analysis of salivary tumor protein p53 (p53) of dogs with benign oral tumors (BN), early- and late-stage oral melanoma (EOM and LOM, respectively), oral squamous cell carcinoma (OSCC) and periodontitis and normal controls (CP); **a** representative western blot for P53 at 53 kDa; **b** bar graph of ratios of P53 protein intensity to total blotted proteins in each lane in a membrane; a-b denote a significant difference at *P* < 0.05; a-c denote a significant difference at *P* < 0.01; a-d denote a significant difference at *P* < 0.001

**Fig. 5:**
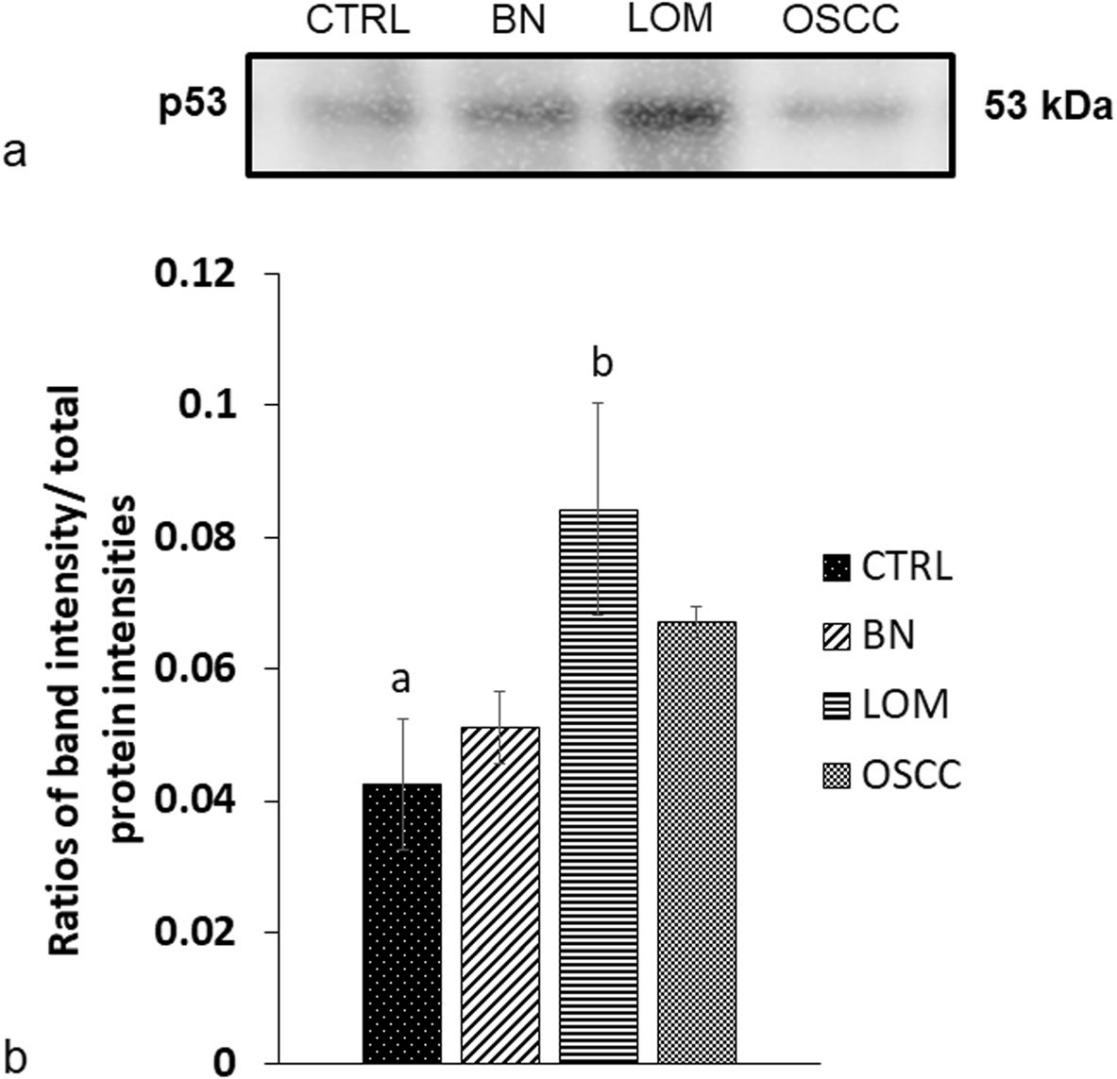
Western blot analysis of tumor protein p53 (p53) in tissues of dogs with benign oral tumors (BN), late-stage oral melanoma (LOM), oral squamous cell carcinoma (OSCC) and normal controls (C); **a** representative western blot for P53 at 53 kDa; **b** bar graph of ratios of P53 protein intensity to total blotted proteins in each lane in a membrane; a-b denote a significant difference at *P* < 0.05

**Fig. 6:**
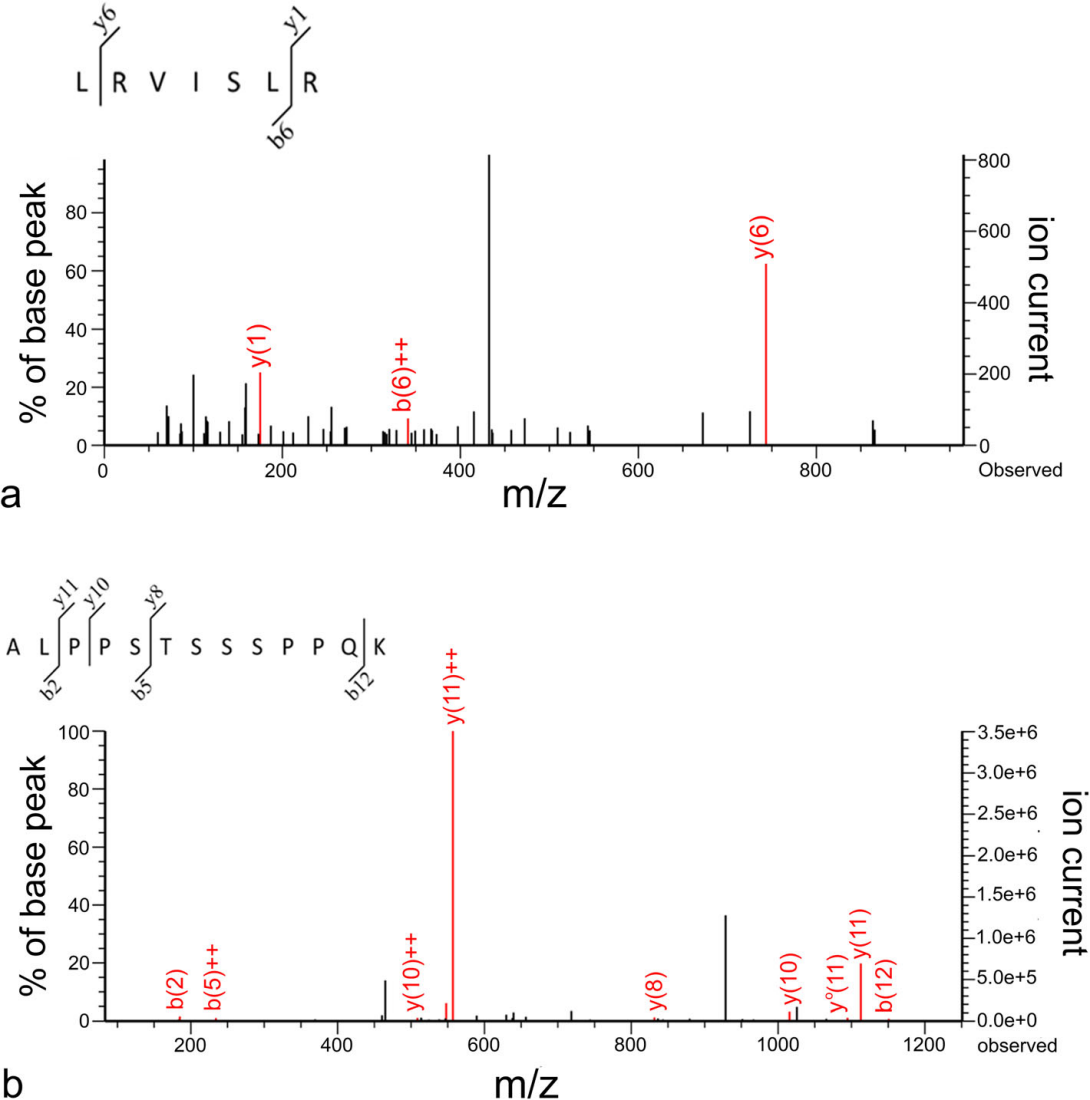
Verification of expressed protein sequences by LC-MS/MS; **a** MS/MS fragmentations of LRVISLR found in salivary tyrosine-protein phosphatase non-receptor type 5 (PTPN5); **b** ALPPSTSSSPPQK found in salivary tumor protein p53 (p53)

Western blot analysis unveiled an enhanced expression of PTPN5 and p53 in saliva of tumor groups compared with that in the CP group (Figs. 3 and 4). In addition, the expression of PTPN5 in LOM and OSCC was augmented compared with that in BN and EOM (Fig. 3). For tissue samples, we did not detect PTPN5 antibody binding to the tissue proteins (Data not shown). For the p53 western blotting, increased expression of p53 was observed in LOM compared with the control group (Fig. 5). Peptide sequences of PTPN5 and p53 western blot analysis were verified by LC-MS/MS (Fig. 6).

## Discussion

In the present study, GeLC-MS/MS was used to identify novel salivary biomarker candidates in canine oral tumors. PTPN5 and p53 were plausibly shown to be candidates in LOM and OSCC. PTP is a group of protein tyrosine phosphatases that have divergent functions, either promoting or suppressing cancer. Several oncogenic PTPs have been reported to be highly expressed in human breast cancer [12]. In contrast to receptor-type PTPs that localized to the plasma membranes, the non-receptor type PTPs, PTPNs, are located in the cytosol. PTPN5 is in the same non-receptor Cys-based classical PTPs as PTPN1 and PTPN11, which promoted tumorigenesis in ovarian cancer, gastric cancer, prostate cancer, breast cancer, leukaemia, colorectal cancer and uveal melanoma [13–19]. PTPN1 has been reported to be increased in canine oral cancer tissues by MALDI-TOF MS plus LC-MS/MS [20]. PTPN1 functioned via Src/Ras/Erk and PI3K/Akt pathways, whereas PTPN11 functioned via EGFR/Ras/MAPK pathways [15, 17, 21–23]. To the best of our knowledge, this study presented for the first time the association of salivary PTPN5 expression and canine oral cancers, particularly LOM and OSCC. Since most families of PTPs served as biomarker targets of several anticancer drugs, including PTPN11, PTPN6 and PTP1B, potential inhibitors of PTPN as candidate anticancer drugs for oral tumors should be investigated [24]. In the present study, we did not observe the expression of PTPN5 in any tissue proteins by western blotting. The plausible explanation included the expression of PTPN5 in saliva was not originated from the tumor tissues while proteins in saliva can be produced from salivary glands or can also be transferred from systemic circulation [25].

In the present study, we also exhibited the enhanced expression of p53, in tumor groups, particularly in saliva of LOM and OSCC and in tissues of LOM group. Likewise, p53 was found in the interaction networks of PTPN5 and the chemotherapy drugs cisplatin and doxorubicin. p53 is a tumor suppressor protein; however, mutant p53 protein has been shown to be a biomarker in several cancers, such as human breast cancer, colorectal cancer, ovarian cancer, oesophageal squamous cell carcinoma, non-small cell lung cancer, and a prognostic marker in breast cancer, oesophageal squamous cell carcinoma, colon cancer, non-small cell lung cancer and B cell lymphoma [26–33]. In human head and neck squamous cell carcinoma, p53 mutation played an important role in tumorigenesis and progression. It has been used not only as a risk and prognostic biomarker, but also as a predictive biomarker in the clinical response to chemotherapy treatments [34–38]. Several studies, aiming to treat cancer in humans, have investigated the promoting function of wild-type p53 and degradation of mutant p53 [29, 39, 40]. Further investigation of p53 in canine oral tumors for potential prognostic and therapeutic biomarkers should be performed.

In the present study, increased expression of another protein involved in the SUMOylation process, RanBP2, was noted in a cancerous group (Table 2). In our previous study of salivary proteomics of canine oral tumors using MALDI-TOF MS and LC-MS/MS, the expression of sentrin-specific protease 7 (SENP7) was found to be increased in saliva of dogs with BN, EOM, LOM and OSCC. And according to the western blot analysis to validate MS results in individual samples, the enhanced expression of SENP7 has been observed in LOM and OSCC, compared with that in CP and BN [6]. SENP7 functions to edit the poly-small ubiquitin-related modifier (SUMO) chains during SUMOylation, a post-translational modification of target proteins involving in several carcinogenic mechanisms [41]. In the present study using the same samples with the previous one, we found the expression of predicted SENP7 (Accession number: XP_008265236.1) in CP, BN, EOM and LOM groups but not in the OSCC group (Additional file 1). And this is probably due to different MS techniques and data analysis methods including different sample preparations, ionization approaches, and statistical analysis [20]. For MALDI-TOF MS coupled with LC-MS/MS, unique PMF peak spectra were previously selected by ClinProTools program before being sequenced by LC-MS/MS. For GeLC-MS/MS, all proteins were loaded into the SDS-PAGE, trypsinized and applied to LC-MS/MS. Proteins was quantitated using DeCyder MS Differential Analysis software, searched against the NCBI mammal database using MASCOT software and grouped by jvenn diagram. And that is the reason why we require traditional protein detection methods such as western blots to confirm the proteomic results.

## Conclusion

The present study used GeLC-MS/MS and western blotting to reveal the potential salivary biomarkers of canine oral tumors, PTPN5 and p53. The network interactions between the candidate proteins and chemotherapy drugs were also demonstrated. For future work, signalling pathways and potential inhibitors of the target proteins should be investigated as potential anticancer drugs for canine oral tumors.

## Methods

### Animals

Saliva samples were recruited from dogs with EOM (*n* = 5), LOM (*n* = 24), OSCC (*n* = 10) and BN (*n* = 11) (age range 7–14 years) whereas tissue samples were taken from 11 LOM, 9 OSCC and 9 BN dogs. Patient characteristics were shown in Tables 3 and 4. Patients were scheduled for surgical operations at the Small Animal Teaching Hospital, Faculty of Veterinary Science, Chulalongkorn University and private animal hospitals. They were diagnosed with no prior history of treatments with chemotherapy and/or radiotherapy. The TNM staging of OM and OSCC were determined according to the WHO, whereby EOM and LOM include stages 1–2 and 3–4, respectively [42, 43]. Regional lymph nodes were examined cytologically for metastasis. Tumor spreading to abdominal organs was checked by an ultrasound examination. Skull-to-abdomen radiography was performed by a Brivo DR-F digital X-ray system (GE Healthcare, Chicago, IL, USA) or an Optima CT660 64-slice CT scanner (GE Healthcare). Seven saliva samples and 10 normal gingival tissue samples were obtained from healthy dogs with no history or clinical signs of oral cavity or cancers (age range 7–8 years). A chronic periodontitis group contained 5 dogs showing gingivitis, dental tartar and/or periodontal attachment loss (age range 7–13 years). The sample collection protocol was approved by the Chulalongkorn University Animal Care and Use Committee (CU-ACUC), Thailand (Approval number 1631042) and written informed consents were obtained from all dog owners.

**Table 3:** Patient characteristics of the saliva of canine samples

Sample no.	Groups^a^	Histological examination	Age (y)	Sex^b^	Breed
1	Control	Normal gingiva	8	F	Beagle
2	Control	Normal gingiva	8	F	Beagle
3	Control	Normal gingiva	8	F	Beagle
4	Control	Normal gingiva	8	F	Beagle
5	Control	Normal gingiva	8	F	Beagle
6	Control	Normal gingiva	8	F	Beagle
7	Control	Normal gingiva	8	F	Beagle
8	Periodontitis	Gingival hyperplasia	10	Mc	Mixed
9	Periodontitis	Gingival hyperplasia	12	Fs	Golden Retriever
10	Periodontitis	Gingival hyperplasia	10	M	Mixed
11	Periodontitis	Gingival hyperplasia	9	M	Pomeranian
12	Periodontitis	Gingival hyperplasia	14	Fs	Shi-tsu
13	Benigh oral tumor	Peripheral odontogenic fibroma	7	Fs	Poodle
14	Benigh oral tumor	Acanthomatous ameloblastoma	10	F	Shi-tsu
15	Benigh oral tumor	Acanthomatous ameloblastoma	11	F	Labrador retriever
16	Benigh oral tumor	Peripheral odontogenic fibroma	10	Mc	Mixed
17	Benigh oral tumor	Peripheral odontogenic fibroma	10	M	Poodle
18	Benigh oral tumor	Peripheral odontogenic fibroma	8	Mc	Siberian husky
19	Benigh oral tumor	Peripheral odontogenic fibroma	10	Fs	Siberian husky
20	Benigh oral tumor	Peripheral odontogenic fibroma	9 Y	M	Shi-Tzu
21	Benigh oral tumor	Peripheral odontogenic fibroma	14 Y	M	Golden Retriever
22	Benigh oral tumor	Peripheral odontogenic fibroma	2 Y	F	Golden Retriever
23	Benigh oral tumor	Acanthomatous ameloblastoma	11	Fs	Golden Retriever
24	OSCC	well differentiated	11	M	Mixed
25	OSCC	well differentiated	13	Fs	Cocker spaniel
26	OSCC	poorly differentiated	9	M	Shi-tsu
27	OSCC	well differentiated	14	Fs	Pug
28	OSCC	poorly differentiated	15	Mc	Poodle
29	OSCC	well differentiated	11	Fs	Poodle
30	OSCC	well differentiated	11	M	Mixed
31	OSCC	poorly differentiated	12	F	Bangkeaw
32	OSCC	well differentiated	12	F	Mixed
33	OSCC	poorly differentiated	11	M	Mixed
34	Early-stage OM (I)	Melanotic melanoma	10	M	Poodle
35	Early-stage OM (I)	Amelanotic melanoma	14	M	Mixed
36	Early-stage OM (II)	Melanotic melanoma	10	Fs	Mixed
37	Early-stage OM (II)	Melanotic melanoma	11	M	chihuahua
38	Early-stage OM (II)	Amelanotic melanoma	12	M	Poodle
39	Late-stage OM (III)	Melanotic melanoma	12	M	Pug
40	Late-stage OM (III)	Melanotic melanoma	12	M	Labrador retriever
41	Late-stage OM (IV)	Melanotic melanoma	14	M	Cocker spaniel
42	Late-stage OM (III)	Melanotic melanoma	8	M	Schnauzer
43	Late-stage OM (III)	Amelanotic melanoma	11	M	Poodle
44	Late-stage OM (III)	Melanotic melanoma	15	M	Shi-tsu
45	Late-stage OM (III)	Melanotic melanoma	13	Fs	Golden Retriever
46	Late-stage OM (III)	Melanotic melanoma	14	M	Mixed
47	Late-stage OM (III)	Melanotic melanoma	13	F	Poodle
48	Late-stage OM (III)	Melanotic melanoma	12	M	Pomeranian
49	Late-stage OM (IV)	Melanotic melanoma	15	M	Golden Retriever
50	Late-stage OM (III)	Amelanotic melanoma	13	M	Cocker spaniel
51	Late-stage OM (III)	Melanotic melanoma	14	M	Golden Retriever
52	Late-stage OM (III)	Melanotic melanoma	12	M	Mixed
53	Late-stage OM (III)	Amelanotic melanoma	10	M	Mixed
54	Late-stage OM (III)	Melanotic melanoma	14	M	Mixed
55	Late-stage OM (III)	Melanotic melanoma	15	M	Poodle
56	Late-stage OM (III)	Melanotic melanoma	8	M	Golden Retriever
57	Late-stage OM (III)	Melanotic melanoma	10	Fs	Beagle
58	Late-stage OM (III)	Amelanotic melanoma	10	M	Mixed
59	Late-stage OM (III)	Amelanotic melanoma	8	M	Mixed
60	Late-stage OM (III)	Amelanotic melanoma	12	Fs	Dachshund
61	Late-stage OM (IV)	Melanotic melanoma	14	M	Poodle
62	Late-stage OM (III)	Melanotic melanoma	12	F	Golden Retriever

Clinical stages are in parentheses

^a^*OM* Oral melanoma, *OSCC* Oral squamous cell carcinoma

^b^*M* Male, *Mc* Male castration, *F* Female, *Fs* Female spray

**Table 4:** Patient characteristics of the canine gingival tissues

Sample no.	Groups^b^	Histological examination	Age (year)	Sex^b^	Breed
1	Control	Normal gingiva	8	F	mixed
2	Control	Normal gingiva	6	M	mixed
3	Control	Normal gingiva	7	M	mixed
4	Control	Normal gingiva	4	Mc	Beagle
5	Control	Normal gingiva	1	F	mixed
6	Control	Normal gingiva	8	F	Beagle
7	Control	Normal gingiva	8	F	Beagle
8	Control	Normal gingiva	8	F	Beagle
9	Control	Normal gingiva	8	F	Beagle
10	Control	Normal gingiva	8	F	Beagle
11	benign oral tumor	Acanthomatous ameloblastoma	8	Fs	Rottweiler
12	benign oral tumor	Acanthomatous ameloblastoma	9	Mc	mixed
13	benign oral tumor	Peripheral odontogenic fibroma	6	Mc	Shi-Tzu
14	benign oral tumor	Acanthomatous ameloblastoma	7	F	Beagle
15	benign oral tumor	Acanthomatous ameloblastoma	8	Fs	Chi hua hua
16	benign oral tumor	Peripheral odontogenic fibroma	9	M	Shi-Tzu
17	benign oral tumor	Peripheral odontogenic fibroma	14	M	Golden Retriever
18	benign oral tumor	Peripheral odontogenic fibroma	2	F	Golden Retriever
19	benign oral tumor	Acanthomatous ameloblastoma	6	M	Mixed
20	OSCC	poorly differentiated	10	F	Mixed
21	OSCC	well differentiated	17	Fs	Shi-Tzu
22	OSCC	poorly differentiated	10	M	Mixed
23	OSCC	well differentiated	3	M	Shi-Tzu
24	OSCC	well differentiated	11	M	Schnauzer
25	OSCC	well differentiated	10	M	mixed
26	OSCC	well differentiated	15	Fs	Miniature pinscher
27	OSCC	well differentiated	10	Mc	mixed
28	OSCC	well differentiated	10	M	Shi-Tzu
29	Late-stage OM (IV)	Amelanotic melanoma	12	Fs	Mixed
30	Late-stage OM (IV)	Melanotic melanoma	13	F	English cocker spaniel
31	Late-stage OM (III)	Melanotic melanoma	10	Fs	Mixed
32	Late-stage OM (III)	Amelanotic melanoma	10	M	Labrador Retriever
33	Late-stage OM (III)	Melanotic melanoma	14	M	Golden Retriever
34	Late-stage OM (III)	Amelanotic melanoma	11	M	Mixed
35	Late-stage OM (III)	Melanotic melanoma	10	Fs	Poodle
36	Late-stage OM (III)	Melanotic melanoma	9	Fs	Rottweiler
37	Late-stage OM (III)	Melanotic melanoma	12	M	Mixed
38	Late-stage OM (III)	Amelanotic melanoma	10	F	Shi-Tzu
39	Late-stage OM (III)	Melanotic melanoma	12	Mc	German Shepherd

Clinical stages are in parentheses

^a^*OM* Oral melanoma, *OSCC* Oral squamous cell carcinoma

^b^*M* Male, *Mc* Male castration, *F* Female, *Fs* Female spray

### Sample collection and preparation

Saliva was collected on the day of surgery without stimulation. Dogs were fasted for at least 1 h and their mouths were rinsed with 0.9% sterile saline solution [9]. Whole saliva (0.5–1.0 mL) was collected for 5–10 min using a sterile cotton swab. After centrifugation at 2600×g for 15 min at 4 °C [44], Halt protease inhibitor cocktail (Thermo Fisher Scientific, Waltham, MA, USA) was added to 200 μL of supernatant and samples were kept at − 20 °C until analysis. Total protein concentrations were determined by the Lowry method, using bovine serum albumin as a protein standard [45]. According to our previous peptide profiles obtained from MALDI-TOF MS data, showing the control and chronic periodontitis in the same cluster, control and chronic periodontitis samples were consequently combined as a CP group [6]. For the tissues, samples were kept in RNALater solution at − 20 °C until use.

### Analysis of salivary peptides by GeLC-MS/MS

Salivary peptides were analysed by GeLC-MS/MS as previously described with some modifications [20]. Briefly, 50 μg of pooled samples in each group (CP, BN, EOM, LOM and OSCC) were mixed with loading buffer [0.5 M dithiothreitol (DTT), 10% w/v SDS, 0.4 M Tris-HCl pH 6.8, 50% v/v glycerol, 0.1 mg/ml Bromophenol Blue] and boiled at 90 °C for 5 min prior to separating on 12.5% SDS-PAGE (Atto, Tokyo, Japan). Gels were fixed using 50% methanol, acetic acid and 37% formaldehyde and stained with silver nitrate solution, before being scanned using a GS-710 scanner (Bio-Rad Laboratories, Benicia, CA, USA) and stored in 0.1% acetic acid. After that in-gel tryptic digestion was performed where protein bands in each lane were divided into 17 segments and chopped into 1 mm^3^ pieces. Gel pieces were dehydrated using 100% acetonitrile (ACN) and dried. Cysteines were reduced and alkylated by 10 mM DTT in 10 mM ammonium bicarbonate and 100 mM iodoacetamide in 10 mM ammonium bicarbonate, respectively, prior to dehydrating twice in 100% ACN. After trypsin digestion in 50 mM NH_4_HCO_3_ (pH 7.8) overnight at 37 °C, peptides were extracted from the gels using 50% ACN in 0.1% formic acid (FA). Pooled samples were submitted to a reversed-phase high performance liquid chromatography (HPLC). The gradient-eluted peptides were analysed using an Ultimate 3000 LC System coupled to an HCTUltra PTM Discovery System (Bruker Daltonics, Bremen, Germany). Peptides were separated on a PepSwift monolithic column (100 μm internal diameter × 50 mm) (Thermo Fisher Scientific). Peptide separation was achieved with a linear gradient at a flow rate of 1000 nL/min from 4% ACN, 0.1% FA to 70% ACN, 0.1% FA for 7.5 min with a regeneration step at 90% ACN, 0.1% FA and an equilibration step at 4% ACN, 0.1% FA. The entire process took 20 min. Peptide fragment mass spectra were acquired in a data-dependent Auto MS mode with a scan range 400–1500 m/z. However, in the case of having more than 5 precursor fragments, peptides would be selected from the MS scan at 200–2800 m/z. CompassXport software (Bruker Daltonics) was used to convert data from LC-MS/MS into the mzXML format. Protein quantitation was performed using DeCyder MS Differential Analysis software (DeCyderMS, GE Healthcare) [46, 47]. The peptide sequences were searched against the NCBI mammal database for protein identification using MASCOT software, version 2.2 (Matrix Science, London, UK) [48]. Database query included taxonomy (mammals), enzyme (trypsin), variable modifications (oxidation of methionine residues), mass values (monoisotopic), protein mass (unrestricted), peptide mass tolerance (1.2 Da), fragment mass tolerance (±0.6 Da), peptide charge state (1+, 2+ and 3+) and maximum number of missed cleavages. Proteins were identified from one or more peptides with an individual MASCOT score corresponding to *P* < 0.05. Proteins were annotated by UniProtKB/Swiss-Prot entries (http://www.uniprot.org/) and classified according to their molecular function, biological process and cellular component using the PANTHER classification system, version 8.1 (www.pantherdb.org/) [49]. Protein list comparison among different sample groups was displayed using jvenn diagram (http://bioinfo.genotoul.fr/jvenn/example.html) [50]. The interaction network of candidate proteins and chemotherapy drugs was explored using the Stitch program, version 5.0 (http://stitch.embl.de/) [51].

### Validation of MS results by western blot analysis

Protein concentrations of pooled saliva and tissue samples were determined by Lowry assay, SDS-PAGE and western blotting as described previously [6, 52]. Briefly, samples (10 μg) were mixed with loading dye, heated and applied to a pre-cast NuPAGE 4–12% (w/v) Bis-Tris gel (Thermo Fisher Scientific) using RunBlue MES Run Buffer (Expedeon, Heidelberg, Germany) at 200 V for 90 min. Protein standard marker was PageRuler prestained protein ladder (molecular weight range 10–180 kDa) (Thermo Fisher Scientific). After that, the proteins were transferred to TranBlot Turbo nitrocellulose membranes (Bio-Rad Laboratories) at 25 V for 14 min using Trans-Blot Turbo 5× transfer buffer (Bio-Rad Laboratories). Detection of total protein band intensities in each lane was performed by a Pierce Reversible Protein Stain Kit for Nitrocellulose Membranes (Thermo Fisher Scientific) according to the manufacturer’s instructions. Blocking non-specific protein binding was achieved by 5% bovine serum albumin (BSA) (GoldBio, St Louis, MO, USA) in Tris-buffered saline containing 0.1% Tween 20 (TBST) at 25 °C overnight. After washing with TBST, primary antibodies diluted at 1:1000 were incubated with a membrane at 4 °C overnight, including mouse monoclonal anti-human PTPN5 or STEP (F-9) (Cat. No. sc-514,678, Santa Cruz Biotechnology, Dallas, TX, USA) and mouse monoclonal anti-human p53 (DO-1) (Cat. No. sc-126, Santa Cruz Biotechnology, Dallas, TX, USA). Membranes were washed with TBST and then incubated with 1:10000 horseradish peroxidase conjugated-rabbit anti-mouse IgG secondary antibody (Abcam, Cambridge, UK) for 1 h at 25 °C. The proteins of interest were visualized with ECL western blotting detection reagents (GE Healthcare). Western blot imaging was performed using a ChemiDoc Touch Imaging System (Bio-Rad Laboratories). Protein bands intensities were analysed by Image Lab 6.0.1 software (Bio-Rad Laboratories). Total protein normalization was performed with the modification of Aldridge et al. (2008) [6, 53]. The ratios of target band intensities to the total proteins in each lane were calculated as previously described [6]. The western blotting was performed in triplicate.

### Verification of expressed protein sequences by LC-MS/MS

LC-MS/MS was utilized to confirm PTPN5 and p53 (or TP53) protein identities as described previously [6]. Briefly, blotting membranes were incubated with Restore Plus Western Blot Stripping Buffer (Thermo Fisher Scientific) for 15 min and washed 4 times with TBST. Protein bands were excised and stored in 10 mM DTT in 10 mM ammonium bicarbonate overnight. Samples were then trypsinized at 37 °C for 3 h and applied to the LC-MS/MS as mentioned above.

### Statistical analysis

ANOVA statistical analysis, incorporated into the DeCyder MS differential analysis software, and MASCOT software, version 2.2 were used to analyse significantly different peptide peak intensities and MASCOT LC-MS/MS scores, respectively. Western blot analysis was performed by ordinary one-way ANOVA with Tukey’s multiple comparisons for PTPN5 and p53. Statistical analyses of protein expression data were conducted using GraphPad Prism, version 8.0.1 (GraphPad Software, La Jolla, CA, USA). Significance was accepted at the *P* < 0.05 level.

## Supplementary information

**Additional file 1.** The relative expression levels of proteins found in normal controls and periodontitis (CP), benign tumors (BN), early-stage oral melanoma (EOM), late-stage oral melanoma (LOM) and oral squamous cell carcinoma (OSCC) as log_2_ intensities.

## Data Availability

The datasets used and/or analysed during the current study are available from the corresponding author on reasonable request.

## Abbreviations

ACN: Acetonitrile
Akt: Protein Kinase B
BN: Benign oral tumors
BSA: Bovine serum albumin
CU-ACUC: The Chulalongkorn University Animal Care and Use Committee
CP: Periodontitis and healthy controls
CT: Computer tomography
CPC: Chromosomal passenger complex
DTT: Dithiothreitol
EGFR: Epidermal growth factor receptor
EOM: Early-stage oral melanoma
Erk: Extracellular-signal-regulated-kinase
FA: Formic acid
GeLC-MS/MS: In-gel digestion coupled with mass spectrometry
HCTUltra: High-capacity ion trap mass spectrometry
HPLC: High performance liquid chromatography
i.d.: Inside diameter
IAA: Iodoacetamide
IgG: Immunoglobulin G
LC: Liquid chromatography
LOM: Late-stage oral melanoma
MALDI-TOF MS: Matrix-assisted laser desorption ionization mass spectrometry
MAPK: Mitogen-activated protein kinase
MES buffer: 2-(*N*-morpholino) ethanesulfonic acid buffer
MS: Mass spectrometry
m/z: Mass per charge ratio
NCBI: National Center for Biotechnology Information
NH_4_HCO_3_: Ammonium bicarbonate
OSCC: Oral squamous cell carcinoma
p53: Tumor protein p53
PI3K: Phosphoinositide-3 kinase
PTM: Post-Translation Modification
PTPN1: Protein tyrosine phosphatase non-receptor type 1
PTPN5: Protein tyrosine phosphatase non-receptor type 5
PTPN6: Protein tyrosine phosphatase non-receptor type 6
PTPN11: Protein tyrosine phosphatase non-receptor type 11
PTP1B: Protein tyrosine phosphatase 1B
RanB2: E3 SUMO-protein ligase RanBP2
Ras: Ras protein
SDS: Sodium dodecyl sulfate
SDS-PAGE: Sodium dodecyl sulfate-polyacrylamide gel electrophoresis
SENP3: SUMO Specific-isopeptidase
SENP7: Sentrin-specific protease 7
Src: Proto-oncogene tyrosine-protein kinase
SUMO: Small ubiquitin-like modifier
TBST: Tris buffered saline buffer containing 0.1% Tween 20
TNM Stage: stages according to their primary sizes and metastatic profile, the tumor, node and metastasis
Topoll: Targeting DNA topoisomerase II
Tris-HCl: Tris hydrochloride
WHO: World Health Organization
